# WhatsApp for Discussing Difficult Cases in Pathology Practice

**DOI:** 10.5146/tjpath.2017.01467

**Published:** 2020-05-15

**Authors:** Beuy Joob, Viroj Wiwanitkit

**Affiliations:** Sanitation,1 Medical Academic Center, Bangkok, Thailand; Honorary Professor, Dr. DY Patil University, Pune, India

**Dear Editor,**

We read the publication named “Usefulness of WhatsApp for Discussing Difficult Cases in Pathology Practice: A Moroccan Experience.” with great interest (1). Bennani and Sekal mentioned that *“Sharing microphotographs of histopathological or cytological cases via WhatsApp is a very easy and fast method to obtain a second opinion in pathology practice and also to discuss difficult cases* (1).” We would like to share our ideas on this report. First, there is no doubt that e-communication is useful in medicine. The application of telemedicine can be helpful in diagnostic pathology. The use of WhatsApp has been proven to be useful for pathology training (2). Nevertheless, there are many issues to be recognized. First, sharing a patient’s data on the network via WhatsApp is an exposure of private data to a third party. This has to be carefully done and there should be ways of guaranteeing the patient’s privacy (3). Protecting the patient’s privacy is necessary. As noted by Saranto et al., lack of patient data privacy can affect patient safety (4). Crane and Gardner noted that it is necessary to have standard guidelines for pathologists who use social media professionally (5). The developed guidelines will help prevent unwanted and unexpected problems due to loss of patient privacy during network communication.
